# Alteration in the Functional Organization of the Default Mode Network Following Closed Non-severe Traumatic Brain Injury

**DOI:** 10.3389/fnins.2022.833320

**Published:** 2022-03-28

**Authors:** Muhammad Riddha Abdul Rahman, Aini Ismafairus Abd Hamid, Nor Azila Noh, Hazim Omar, Wen Jia Chai, Zamzuri Idris, Asma Hayati Ahmad, Diana Noma Fitzrol, Ab. Rahman Izaini Ghani Ab. Ghani, Wan Nor Azlen Wan Mohamad, Mohamed Faiz Mohamed Mustafar, Muhammad Hafiz Hanafi, Mohamed Faruque Reza, Hafidah Umar, Mohd Faizal Mohd Zulkifly, Song Yee Ang, Zaitun Zakaria, Kamarul Imran Musa, Azizah Othman, Zunaina Embong, Nur Asma Sapiai, Regunath Kandasamy, Haidi Ibrahim, Mohd Zaid Abdullah, Kannapha Amaruchkul, Pedro Valdes-Sosa, Maria Luisa-Bringas, Bharat Biswal, Jitkomut Songsiri, Hamwira Sakti Yaacob, Putra Sumari, Paramjit Singh Jamir Singh, Azlinda Azman, Jafri Malin Abdullah

**Affiliations:** ^1^Department of Neurosciences, School of Medical Sciences, Universiti Sains Malaysia, Kota Bharu, Malaysia; ^2^School of Medical Imaging, Faculty of Health Sciences, Universiti Sultan Zainal Abidin, Kuala Nerus, Malaysia; ^3^Brain and Behavior Cluster, School of Medical Sciences, Universiti Sains Malaysia, Kota Bharu, Malaysia; ^4^Hospital Universiti Sains Malaysia, Universiti Sains Malaysia, Kota Bharu, Malaysia; ^5^Faculty of Medicine and Health Sciences, Universiti Sains Islam Malaysia, Nilai, Malaysia; ^6^Department of Physiology, School of Medical Sciences, Universiti Sains Malaysia, Kota Bharu, Malaysia; ^7^Department of Community Medicine, School of Medical Sciences, Universiti Sains Malaysia, Kota Bharu, Malaysia; ^8^Department of Paediatrics, School of Medical Sciences, Universiti Sains Malaysia, Kota Bharu, Malaysia; ^9^Department of Ophthalmology, School of Medical Sciences, Universiti Sains Malaysia, Kota Bharu, Malaysia; ^10^Department of Radiology, School of Medical Sciences, Universiti Sains Malaysia, Kota Bharu, Malaysia; ^11^Gleneagles Hospital Kuala Lumpur, Kuala Lumpur, Malaysia; ^12^School of Electrical and Electronic Engineering, Universiti Sains Malaysia, Nibong Tebal, Malaysia; ^13^Graduate School of Applied Statistics, National Institute of Development Administration (NIDA), Bangkok, Thailand; ^14^The Clinical Hospital of Chengdu Brain Science Institute, MOE Key Lab for Neuroinformation, University of Electronic Science and Technology of China, Chengdu, China; ^15^The Cuban Neurosciences Center, Havana, Cuba; ^16^Department of Biomedical Engineering, New Jersey Institute of Technology, Newark, NJ, United States; ^17^EE410 Control Systems Laboratory, Department of Electrical Engineering, Faculty of Engineering, Chulalongkorn University, Bangkok, Thailand; ^18^Department of Computer Science, Kulliyah of Information and Communication Technology, International Islamic University Malaysia, Kuala Lumpur, Malaysia; ^19^School of Computer Sciences, Universiti Sains Malaysia, Gelugor, Malaysia; ^20^School of Social Sciences, Universiti Sains Malaysia, Gelugor, Malaysia

**Keywords:** default mode network, traumatic brain injury, functional connectivity, effective connectivity, neuropsychology

## Abstract

The debilitating effect of traumatic brain injury (TBI) extends years after the initial injury and hampers the recovery process and quality of life. In this study, we explore the functional reorganization of the default mode network (DMN) of those affected with non-severe TBI. Traumatic brain injury (TBI) is a wide-spectrum disease that has heterogeneous effects on its victims and impacts everyday functioning. The functional disruption of the default mode network (DMN) after TBI has been established, but its link to causal effective connectivity remains to be explored. This study investigated the differences in the DMN between healthy participants and mild and moderate TBI, in terms of functional and effective connectivity using resting-state functional magnetic resonance imaging (fMRI). Nineteen non-severe TBI (mean age 30.84 ± 14.56) and twenty-two healthy (HC; mean age 27.23 ± 6.32) participants were recruited for this study. Resting-state fMRI data were obtained at the subacute phase (mean days 40.63 ± 10.14) and analyzed for functional activation and connectivity, independent component analysis, and effective connectivity within and between the DMN. Neuropsychological tests were also performed to assess the cognitive and memory domains. Compared to the HC, the TBI group exhibited lower activation in the thalamus, as well as significant functional hypoconnectivity between DMN and LN. Within the DMN nodes, decreased activations were detected in the left inferior parietal lobule, precuneus, and right superior frontal gyrus. Altered effective connectivities were also observed in the TBI group and were linked to the diminished activation in the left parietal region and precuneus. With regard to intra-DMN connectivity within the TBI group, positive correlations were found in verbal and visual memory with the language network, while a negative correlation was found in the cognitive domain with the visual network. Our results suggested that aberrant activities and functional connectivities within the DMN and with other RSNs were accompanied by the altered effective connectivities in the TBI group. These alterations were associated with impaired cognitive and memory domains in the TBI group, in particular within the language domain. These findings may provide insight for future TBI observational and interventional research.

## Introduction

Traumatic brain injury (TBI) is one of the most common causes of debilitating neurodegenerative diseases that affect more than 10 million people each year globally (Humphreys et al., 2013; Moreno-López et al., 2016). TBI most commonly affects people who are in their productive years, therefore incurring significant economic losses. In addition, TBI also puts a burden on the public healthcare system, as TBI survivors often require assistance and hardly return to a quality life (Majdan et al., 2017). Therefore, research into how TBI affects the functions of the human brain is crucial to understanding the mechanism of injury and how they can be prevented to help manage the TBI survivors to return to the quality of life.

The debilitating effect of TBI can range from mild cognitive disruption to adverse reduction in brain function, depending on the severity of the injury. In severe TBI, the deleterious effects on the brain were more pronounced in more severe cases (Khanmohammadi et al., 2018). As for mild TBI, they are often misdiagnosed (Palacios et al., 2017; Vergara et al., 2018), thus risks being left untreated. This is concerning because the effect of mild TBI can be harmful to the integrity of the brain function and increase the risk of neurodegenerative diseases later in life, however, small the initial concussion might be (Vergara et al., 2018). Therefore, all trauma to the head must receive a proper diagnosis and the integrity of the brain function assessed properly to prevent premature neurodegenerative diseases in TBI survivors.

In achieving this, the resting-state fMRI (rsfMRI) is an indispensable tool to study the extent of functional alterations caused by TBI. Since its inception in 1995, rsfMRI studies have been conducted increasingly to study the brain networks that emerged from seemingly resting conditions; among them is the default mode network (DMN) (Nakamura et al., 2009). The resting-state paradigm is relatively easier to conduct, requiring no explicit tasks and able to accommodate a wide range of participants across all levels of consciousness and cognitive abilities.

The total force of trauma to the head often disrupted the structural integrity of the brain in the form of axonal injury, thus affecting functional connectivity (FC) and cognitive performance (Sours et al., 2017; Gordon et al., 2018; Wooten et al., 2019). However, damaged structural tracts may create juxtaposed effects toward FCs, in which it becomes increased especially involving network hubs (Hillary et al., 2014). Researchers attribute this paradox as the compensatory effect orchestrated by the brain, mainly to cope with inefficient information transfer due to the recruitment of longer tracts (Porter et al., 2017; Wooten et al., 2019). This functional hyperconnectivity is often resolved longitudinally, as the brain finds the balance between optimal performance and network costs (Hillary and Grafman, 2017). Nevertheless, cases of diminished FCs due to trauma were also reported, especially in the earlier stages of TBI (Manning et al., 2019).

The FCs can be illustrated as the statistical connections between cerebral signals across time, which may be used to draw inferences about functional interactions between two or more brain areas. On the other hand, there is another type of brain connectivity termed effective connectivity (EC) which seeks to describe causal links through experimental paradigms or models rather than just looking at correlations between brain activity (Gaudet et al., 2020). This enables the direction of interactions between various brain areas to be deduced.

In this study, we examined the brain responses in healthy controls (HC) and TBI groups and compare them to find any significant difference in the functional organization of the resting-state network, specifically the DMN. Specifically, we investigated the group activations modeled after the low-frequency fluctuation of the brain and compare them to look for significant differences that may account for the TBI effects. In addition, we also analyzed the FCs of both groups to see any changes. Finally, we also correlated the FCs with their performance in psychological tests.

## Materials and Methods

### Participants

Nineteen non-severe TBI participants (mean age 30.84 ± 14.56) were recruited from the emergency department, Hospital Universiti Sains Malaysia. Twenty-two matching controls (mean age 27.23 ± 6.32) were also recruited. All participants were right-handed Malay males aged between 18 and 65 years. All TBI participants sustained non-severe TBI, measured using the Glasgow Coma Scale of between 8 and 15, and scanned at the subacute phase (4-6 weeks) of the injury. The exclusion criteria include any previous TBI history, psychiatric illness, history of drug abuse, ocular injuries, and contraindications to MRI. Participants also gave written consent before being enrolled in the study. The study protocol and procedures were approved by the Institutional Ethics Committee (IEC) of Universiti Sains Malaysia (IEC Code: USM/JEPeM/15110485 and USM/JEPeM/20080406) and carried out under the latest version of the Declaration of Helsinki.

### Neuropsychological Assessment

A subset of the HC and TBI participants was cognitively assessed using neuropsychological tests that comprised the Wechsler Abbreviated Scale of Intelligence 1st edition (WASI; block design and matrix reasoning) to estimate general cognitive ability (McCrimmon and Smith, 2013), Rey Auditory Verbal Learning Test (RAVLT; immediate and delayed recall) to assess the verbal memory function (Bean, 2011; Khosravi Fard et al., 2016), Rey Complex Figure Test and Recognition Trial (RCFT; immediate and delayed recall) to assess the visual memory and perception (Sargénius et al., 2017), Comprehensive Trail-Making Test (CTMT) to assess the psychomotor speed and cognitive flexibility (Gray, 2006; Beratis et al., 2018), and Wisconsin Card Sorting Test (WCST) to measure the executive functioning (Kolakowsky-Hayner, 2011). These tests were appropriate to assess the cognitive domains that are often impaired following TBI. The results are calculated based on the standard scores that are corrected for age and education level. In total, thirteen HC and sixteen TBI participants took part in the neuropsychological tests.

### Magnetic Resonance Imaging Scanning Parameters

The structural and functional MRI data were obtained using a 3.0-T MRI machine (Philips Achieva, Best, The Netherlands) equipped with a 32-channel head coil. The structural images were acquired using T1-weighted imaging, with a Magnetisation Prepare Gradient Echo (MPRAGE) sequence, a 256 × 256 matrix, and 160 sagittal slices. The structural repetition time (TR) was set at 2,000 ms, echo time (TE) was set at 30 ms, flip angle (FA) was set at 8°, and the final resolution of structural images was 1 mm × 1 mm × 1 mm.

Functional images were obtained using the T2* echo planar imaging (EPI) sequence, with a 96 × 96 matrix size and 32 oblique slices, set parallel to the orbitofrontal cortex to reduce the sinus artifact. The TR was set at 1,700 ms, TE at 33 ms, and FA at 78°. The field of view of functional images was set at 192 mm^2^ with slice thickness set at 3 mm with a 0-mm gap. The slice acquisition was interleaved, and a total of 250 scans were obtained in 7 min. During the scanning, participants were asked to close their eyes and remain still without any mental task engagement.

### Data Preprocessing

The acquired fMRI data underwent anonymization and converted from DICOM to NIFTI format for subsequent data analysis. Before preprocessing, the first ten volumes of the data were removed to avoid the initial MRI signal instability and account for participants’ adaptation to the scanner (Li et al., 2019). Data were preprocessed using Statistical Parametric Mapping 12 (SPM12)^1^ software package implemented in MATLAB (v. R2021a)^2^ using a standard preprocessing pipeline. First, functional data underwent slice timing correction and realignment to correct the motion artifact and then co-registered to individual T1 anatomical images. The anatomical images were then fitted into standard space according to Montreal Neurological Institute (MNI), and the normalized parameters were applied to the functional data. Lastly, a Gaussian blur set at 8 mm full width half maximum (FWHM) was applied to the functional data to obtain better inference of the neighboring voxels. Artifact Detection Toolbox (ART)^3^ implemented in MATLAB was used to detect any outlier scans. The global mean threshold was set at a 3 standard deviation (SD) limit with a movement threshold of 0.5 mm (Dailey et al., 2018), corresponding to a conservative 95% confidence interval.

### Statistical Analysis

Demography, neuropsychological scores, and correlational analysis were carried out using Statistical Product and Service Solutions (SPSS 26). The results of demography and neuropsychological data were compared between groups using an independent-sample t-test with a p-value set at 0.05, and Levene’s test for assumption of equal variance was conducted simultaneously.

In addition to statistical analysis, effect size calculations were also performed to compare the effect of the sample size of each group. Due to the difference in group size, corrected Cohen’s d (Hedge’s g) was used to estimate the effect size and aid in result interpretation (Gerchen et al., 2021; Pernet et al., 2021). Following the rule of thumb set by Cohen, an effect size of 0.2 is considered as a small effect, 0.5 as a medium effect, and 0.8 as a large effect (Lakens, 2013).

### Low-Frequency Fluctuations Modeling

The general linear model (GLM) was designed according to the steps outlined in the technical paper by Di and Biswal (2014). The low-frequency fluctuations (LFF) were modeled into eight-block functions that represented the following frequencies: 0.01, 0.02, 0.04, and 0.08 Hz, with a 90° offset for each frequency (Di and Biswal, 2014). Preprocessed data of each participant were entered into the GLM and subjected to one-sample t-tests to obtain the activation of the brain areas. The peak voxel activations were thresholded at *p* < 0.001 (uncorrected, p_*unc*_), while cluster size activation was adjusted to correct for false discovery rate (FDR) at *p*-value < 0.05 (p_*FDR*_) (Sours et al., 2017). Afterward, the GLMs from each group were entered into 2nd-level analysis to find any significant differences in the activation patterns. For this purpose, a two-sample t-test was used with similar peak voxel and cluster size activation threshold (p_*unc*_ < 0.001 and p_*FDR*_ < 0.05, respectively).

### Functional Connectivity Analysis

The FC analysis was conducted using CONN Toolbox (v.20b; RRID: SCR_009550)^4^, open-source software based on MATLAB/SPM12 for FC analysis of fMRI data (Whitfield-Gabrieli and Nieto-Castanon, 2012). Preprocessed images underwent denoising step, which removes signals from the white matter and cerebrospinal fluids and discards outlier scans caused by motion artifacts. In addition, a temporal bandpass filtering was applied to account for the LFF, set between 0.009 and 0.08 Hz.

Following that, seed-based and region of interest (ROI)-based FC analyses were performed. The DMN consists of four nodes indicated *a priori* by the CONN Toolbox. The nodes included the medial prefrontal cortex (MPFC), posterior cingulate cortex (PCC), and left and right lateral parietal (LP), outlined in Table 1. For the ROI-to-ROI analysis, 32 nodes from 8 resting-state networks were set based on the CONN network *a priori* groups implemented in CONN Toolbox as follows: DMN (4 nodes), sensorimotor network (SN; 3 nodes), visual network (VN; 4 nodes), salience network (SN; 7 nodes), dorsal attention network (DAN; 4 nodes), frontoparietal network (FPN; 4 nodes), language network (LN; 4 nodes), and cerebellar network (CN; 2 nodes). Similar to LFF modeling, multiple comparisons in the cluster level were corrected using p_*FDR*_ < 0.05 and voxel-level threshold set at p_*unc*_ < 0.001 (Sours et al., 2017).

**TABLE 1:** Functionally defined nodes of the default mode network as outlined in the CONN Toolbox.

Nodes	Central coordinate		
Medial prefrontal cortex	1	55	−3
Posterior cingulate cortex	1	−61	38
L lateral parietal	−39	−77	33
R lateral parietal	47	−67	29

*R: right, L: left. Coordinates follow the standard Montreal Neurological Institute (MNI) template in millimeters (mm).*

### Dynamic Causal Modeling

We analyzed the ECs of the DMN using Dynamic Causal Modeling embedded within the SPM12 (DCM10.5). The LFF signal from four nodes of the DMN, precuneus, MPFC, and bilateral angular gyrus was extracted from the *a priori* DMN nodes specified in the CONN Toolbox previously. For this purpose, we set a sphere of 8-mm radius as the volume of interest centered on the peak coordinates of each node. In the analysis, the LFF signals of these DMN nodes were regressed against the LFF signals from white matter and cerebrospinal fluids to remove any effects that may be contributed by these components.

**FIGURE 1:**
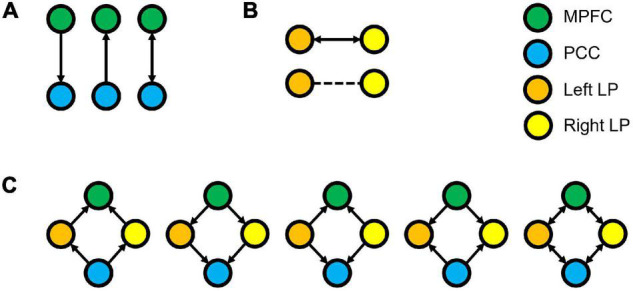
Three families of the DCM models, where **(A)** denotes the possible connectivity directions between MPFC and PCC, **(B)** denotes the possible connectivity directions between the LLP and RLP, and **(C)** denotes the possible connectivity directions between MPFC, PCC, LLP, and RLP. The combination of these families yielded 30 possible DCM models that were compared to find the winning model.

The endogenous connectivities between these nodes were modeled following the methods outlined by Di and Biswal (2014), in which the connectivity was varied between three possible combination families: PCC–MPFC, LLP–RLP, and PCC/MPFC–LLP/RLP, as outlined in Figure 1 (Di and Biswal, 2014). The resulting number of models was subsequently analyzed using cross-spectral density, as resting-state data are appropriately analyzed using the frequency domain. All models were compared using Bayesian model selection (BMS) using random-effect inference to determine the best model. Random-effect BMS is favorable as it is impervious to outliers, thus ensuring group heterogeneity (Sadeghi et al., 2020). The probability graph of each model was plotted, and the winning model was selected according to the maximum probability among all the models. The winning model from each group was then averaged using Bayesian Parameter Averaging, a method of integrating the individual posterior densities and utilizing the posterior from one subject as the prior for the successive subject (Stephan et al., 2010).

## Results

### Demographics and Neuropsychological Results

The demographic and neuropsychological information and results are detailed in Table 2. Our cohort of participants consisted of a homogenous sample in terms of race and gender (Malay males). Sixteen TBI and thirteen HCs from the sample size were administered the neuropsychological tests. The independent t-test revealed no significant difference between TBI and HC in the WASI, RAVLT, RCFT, CTMT, and WCST domains. However, medium effect sizes were observed on the matrix reasoning domain in WASI (t[27] = 1.77, *p* = 0.089, Hedge’s *g* = 0.66), both domains of the RAVLT (immediate verbal recall, t[27] = 1.60, *p* = 0.122, Hedge’s *g* = 0.60; delayed verbal recall, t[27] = 1.33, *p* = 0.194, Hedge’s *g* = 0.50), and the immediate recall domain in RCFT (t[27] = 1.74, *p* = 0.093, Hedge’s *g* = 0.65), which indicated medium practical differences. These results suggest that the TBI group performed moderately worse than HC in general cognitive ability, verbal memory, and visual memory. Other domains and tests recorded either small or trivial effect sizes.

**TABLE 2:** Demographics and neuropsychological characteristics.

		Mean HC (SD)	Mean TBI (SD)	*t*	p^2^	Effect size*^b^*
Age		27.23 (6.32)	30.84 (14.56)	−-1.00	0.326	0.33
Education (HC: *n* = 13, TBI: *n* = 17)		15.54 (1.94)	12.59 (2.83)	3.22	0.003	1.19
Days since injury		−	40.63 (10.14)	−	−	−
GCS (median)		−	15	−	−	−
WASI (HC: *n* = 13, TBI: *n* = 16)						
	Block design	51.69 (9.04)	49.69 (6.18)	0.708	0.485	0.26
	Matrix reasoning	51.08 (10.61)	43.38 (12.48)	1.765	0.089	0.66
RAVLT (HC: *n* = 13, TBI: *n* = 16)						
	Immediate recall	46.77 (9.05)	40.75 (10.88)	1.595	0.122	0.60
	Delayed recall	10.08 (2.78)	8.31 (4.06)	1.331	0.194	0.50
RCFT (HC: *n* = 13, TBI: *n* = 16)						
	Immediate recall	52.38 (11.01)	42 (19.03)	1.742	0.093	0.65
	Delayed recall	47.08 (14.67)	40.63 (17.53)	1.059	0.299	0.40
CTMT (HC: *n* = 13, TBI: *n* = 16)		85.08 (21.64)	84.13 (17.32)	0.132	0.896	0.05
WCST (HC: *n* = 13, TBI: *n* = 16)		37.15 (10.39)	32.69 (15.41)	0.892	0.38	0.33

*WASI, Wechsler Abbreviated Scale of Intelligence; RAVLT, Rey Auditory Verbal Learning Test; RCFT, Rey Complex Figure Test and Recognition Trial; CTMT, Comprehensive Trail-Making Test; WCST, Wisconsin Card Sorting Test; HC, Healthy control; TBI, traumatic brain injury; SD, standard deviation.*

*^a^Two-tailed independent sample t-test at p < 0.05.*

*^b^Cohen’s corrected d (Hedge’s g).*

### Brain Structural Evaluations

The T1-weighted structural images were evaluated independently by three senior neurosurgeons with more than 15 years of experience from the Department of Neurosciences, Hospital Universiti Sains Malaysia. No significant structural alterations were identified in the brain morphometry.

### Low-Frequency Fluctuations Activations Comparison

**FIGURE 2:**
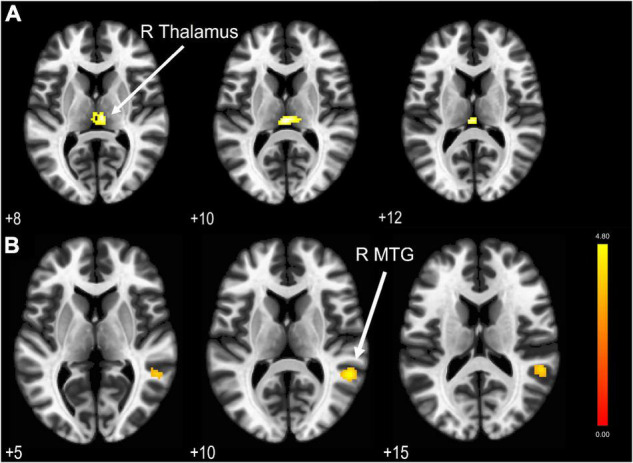
**(A)** LFF BOLD activation comparison revealed a significant hypoactivation area in the TBI group compared to HC with cluster extent threshold *k* = 79. **(B)** Significantly activated areas in the TBI group (correspond to the middle temporal gyrus) due to hypoconnectivity from PCC as the seed region. Results are thresholded at voxel-level p_*unc*_ < 0.001 and cluster extent thresholded at p_*FDR*_ < 0.05. The color bar represents the T-score.

We compared the BOLD activations to find any difference in the LFF between HC and TBI groups. The independent sample t-test showed no significant activations when we applied the cluster-level threshold at p_*FDR*_ < 0.05. Nevertheless, by using the lower cluster threshold (p_*unc*_ < 0.05), we found a significant hypoactivation (see Figure 2A) in the TBI group at the right thalamus (peak MNI coordinate 0 -24 10, t[39] = 4.54, *p* < 0.05). Results are outlined in Table 3.

**TABLE 3:** LFF statistical analysis with significantly hypoactivated areas in the TBI group compared to the HC.

Area	Coordinate (*x y z*)			Cluster size*^a^*	*T*
R thalamus	0	−24	10	79	4.54

*R, right. Coordinates follow the standard Montreal Neurological Institute (MNI) template in millimeters (mm).*

*^a^p_FDR_ < 0.05.*

### Functional Connectivity in Healthy Controls and Traumatic Brain Injury Groups

We computed seed-based and ROI-based analyses to measure the FC in both HC and TBI. In essence, the seed-based connectivity measures the connectivity of a seed region to other areas of the brain, while ROI-based connectivity compares the parameter of connectivity between different regions of interest associated with a particular network. The parameters of each participants’ FC were then compared between HC and TBI.

### Seed-Based Analysis

The four DMN nodes that were set as seeds are outlined in Table 1. Figure 2B shows the result of seed-based analysis of the DMN nodes, which reveals a significantly reduced FC in the TBI group between the PCC and middle temporal gyrus (MTG; peak MNI coordinate 54 -46 10, cluster-level p_*FDR*_ = 0.013, Hedge’s *g* = 0.79). The result is outlined in Table 4.

**TABLE 4:** The regions that displayed significant activation in the HC > TBI, based on seed regions of individual DMN nodes.

Seed	Activated regions	Coordinate (*x y z*)			Cluster size	p*^a^*	Effect size*^b^*
PCC	Middle temporal gyrus	54	−46	10	183	0.013	0.79

*PCC, posterior cingulate cortex. Coordinates follow the standard Montreal Neurological Institute (MNI) template in millimeters (mm).*

*^a^p_FDR_ < 0.05.*

*^b^Cohen’ s corrected d (Hedge’s g).*

### Region of Interest -Based Analysis

**FIGURE 3:**
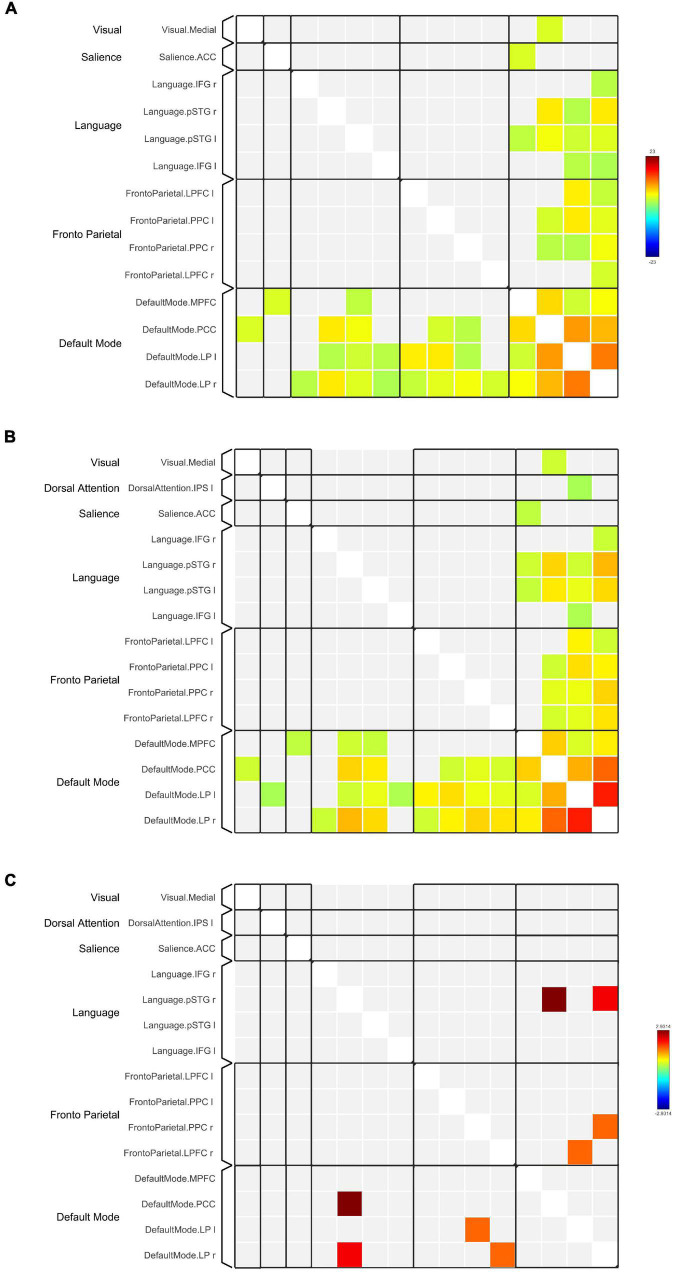
Functional connectivity matrices within the DMN and between the DMN and other RSNs in **(A)** HC and **(B)** TBI groups. Results in **(A,B)** are thresholded at voxel-level p_*unc*_ < 0.001 and cluster extent thresholded at p_*FDR*_ < 0.05, corrected for multiple comparisons. **(C)** The 2-sample *t*-test between HC and TBI groups show functional connectivity matrices that achieve moderate to high effect size. The color bars represent the T-score.

Network-based F-statistics analysis in eight *a priori* RSNs revealed no significant difference in FC between groups at network level p_*FDR*_ < 0.05. The FC matrices are presented in Figures 3A,B. However, the independent t-test for individual nodes FC reveals significant hypoconnectivity in ROI pairs between DMN and LN in the TBI group, in PCC and right posterior superior temporal gyrus (pSTG) (t[39] = 2.93, *p* = 0.006, Hedge’s *g* = 0.92), and right LP and right pSTG (t[39] = 2.26, *p* = 0.029, Hegde’s *g* = 0.71). Additionally, the effect size estimates for two ROI pairs between DMN and FPN indicated to have potential medium practical differences, thus suggesting that the TBI group was hypoconnected between left LP and right lateral prefrontal cortex (LPFC) (t[39] = 1.63, *p* = 0.112, Hedge’s *g* = 0.51) and between right LP and right posterior parietal cortex (PPC) (t[39] = 1.56, *p* = 0.126, Hedge’s *g* = 0.29). The results are outlined in Table 5, and the FC matrices are presented in Figure 3C. In addition, based on our findings in LFF activation, we analyzed the FC between DMN nodes and thalamus. We did not find any significant difference in terms of FC between the HC and TBI. Moreover, the effect size is negligible.

**TABLE 5:** The effect size of the ROI pairs between HC and TBI groups that achieve moderate to high effect as per Cohen’s recommendations.

Inter-DMN connectivity	ROI pair	Mean HC (SD)	Mean TBI (SD)	*t*	p^2^	Effect size*^b^*
DMN–LN	PCC-R pSTG	0.42 (0.22)	0.24 (0.16)	2.93	0.006*	0.92
DMN–LN	R LP-R pSTG	0.49 (0.27)	0.32 (0.21)	2.26	0.029*	0.71
DMN–FPN	L LP-R LPFC	0.20 (0.23)	0.08 (0.23)	1.63	0.112	0.51
DMN–FPN	R LP-R PPC	0.46 (0.29)	0.33 (0.28)	1.56	0.126	0.49

*R, right; L, left; pSTG, posterior superior temporal gyrus; LP, lateral parietal; LPFC, lateral prefrontal cortex; PPC, posterior parietal cortex; DMN, default mode network; LN, language network; FPN, fronto-parietal network.*

*^a^Two-tailed independent-sample t-test.*

*^b^Cohen’s corrected d (Hedge’s g).*

*p < 0.05.

### Correlation Between Neuropsychological Performance and Default Mode Network Connectivity

We performed correlation analysis between the neuropsychological performance and the FC in the TBI group. Our results suggested that the alterations in the FC within and between the DMN and other RSNs formed significant associations with the scores of the neuropsychology assessment. We found that higher scores in the following tests are significantly correlated with higher FC: (1) T-block with PCC–left pSTG (R = 0.63, *p* = 0.008) and left LP–right LP (R = 0.63, *p* = 0.009); (2) verbal memory with PCC–left pSTG (immediate recall R = 0.57, *p* = 0.022; delayed recall R = 0.53, *p* = 0.034), left LP–right LP (immediate recall R = 0.054, *p* = 0.031; delayed recall R = 0.511, *p* = 0.043), and left LP–left IFG (delayed recall R = 0.52, *p* = 0.041); and 3) CTMT with PCC–right pSTG (R = 0.50, *p* = 0.048), PCC–right PPC (R = 0.58, *p* = 0.019), PCC–right LPFC (R = 0.54, *p* = 0.032), and right LP–right IFG (R = 0.61, *p* = 0.012). We also found that higher scores in the following tests are significantly correlated with lower FC: (1) matrix reasoning with PCC–VN (R = −0.51, *p* = 0.042); (2) visual memory with PCC–VN (immediate recall R = −0.56, *p* = 0.026; delayed recall R = −0.58, *p* = 0.020); and 3) CTMT with MPFC–PCC (R = −0.51, *p* = 0.045). The results are outlined in Table 6 (correlation graphs are available in Supplementary Material).

**TABLE 6:** The correlation between the ROI connectivity values and neuropsychological scores in the TBI group.

Domain		Inter-DMN FC	ROI pair	R	p^2^
WASI					
	Block design	DMN–DMN	L LP–R LP	0.63	0.009
		DMN–LN	PCC–L pSTG	0.63	0.008
	Matrix reasoning	DMN-VN	PCC–Visual medial	−0.51	0.042
RAVLT					
	Immediate	DMN–DMN	L LP–R LP	0.54	0.031
		DMN–LN	PCC–L pSTG	0.57	0.022
	Delayed	DMN–DMN	L LP–R LP	0.51	0.043
		DMN–LN	L LP–L IFG	0.52	0.041
		DMN–LN	PCC–L pSTG	0.53	0.034
RCFT					
	Immediate	DMN–VN	PCC–visual medial	−0.56	0.026
	Delayed	DMN–VN	PCC–visual medial	−0.58	0.020
CTMT		DMN – DMN	MPFC-PCC	−0.51	0.045
		DMN-LN	PCC–R pSTG	0.50	0.048
		LN	R LP–R IFG	0.61	0.012
		FPN	PCC–R PPC	0.58	0.019
		FPN	PCC–R LPFC	0.54	0.032

*R, right; L, left; LP, lateral parietal; PCC, posterior cingulate cortex; pSTG, posterior superior temporal gyrus; IFG, inferior frontal gyrus; MPFC, medial prefrontal cortex; PPC, posterior parietal cortex; LPFC, lateral prefrontal cortex; WASI, Wechsler Abbreviated Scale of Intelligence; RAVLT, Rey Auditory Verbal Learning Test; RCFT, Rey Complex Figure Test and Recognition Trial; CTMT, Comprehensive Trail-Making Test; WCST, Wisconsin Card Sorting Test; DMN, default mode network; LN, language network; VN, visual network; FPN, fronto-parietal network.*

*^a^p < 0.05.*

### Effective Connectivity Following Traumatic Brain Injury

**FIGURE 4:**
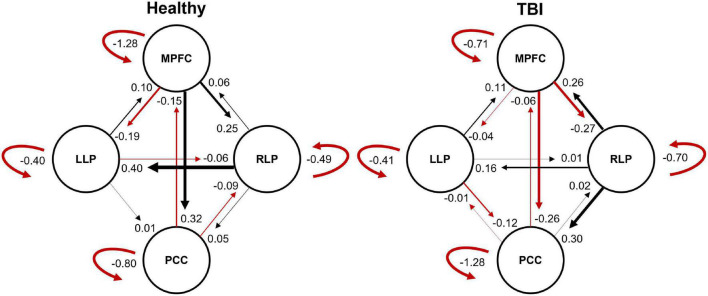
The endogenous connectivity parameters for HC and TBI groups. The effective connectivity between PCC and LLP did not survive statistical significance for the HC group. The thickness of the arrow represents the strength (Hz) of the connection. The black arrow denotes positive connectivity value, which suggests excitation, and the red arrow denotes negative connectivity value, which suggests inhibition.

**FIGURE 5:**
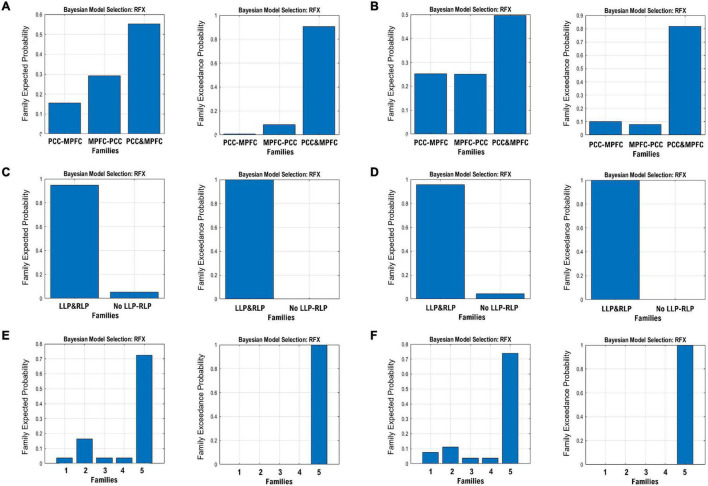
The expected probability (left) and exceedance probability (right) for the three DCM families specified in Figure 1. The BMS results are shown for the MPFC-PCC family in the **(A)** HC group and **(B)** TBI group, LLP-RLP families in the **(C)** HC group and **(D)** TBI group, and bilateral LP and PCC/MPFC families in the **(E)** HC group and **(F)** TBI group.

**FIGURE 6:**
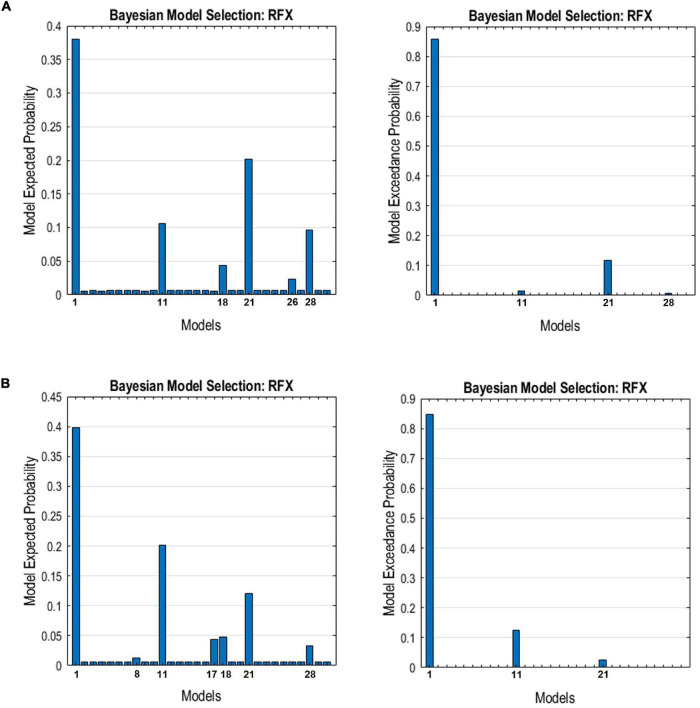
The expected probability (left) and exceedance probability (right) for the individual models compared using Bayesian model selection for the DMN connectivity models in the **(A)** HC group and **(B)** TBI group.

The resulting ECs are presented as either excitatory or inhibitory, characterized by positive and negative values, respectively. Two types of ECs are reported: the self-connection or intrinsic EC, and outgoing connections or extrinsic ECs. The detailed statistical results are presented in Table 7, while Figure 4 visualizes the endogenous ECs in both HC and TBI. After comparing all possible models using random-effect BMS, we found that the full connectivity model is the most optimal model for both HC and TBI groups, in which all regions mutually influence each other (see Figures 5, 6). While the full connectivity model was favorable in the HC group, the connectivity between the left LP and the PCC did not achieve statistical significance. This left the HC group with eleven extrinsic ECs versus twelve in the TBI group. Out of the twelve extrinsic connections, seven ECs in the TBI group are inhibitory (PCC → MPFC, PCC → LLP, MPFC → PCC, MPFC → LLP, MPFC → RLP, LLP → PCC, and LLP → RLP), compared to five in HC (PCC → MPFC, PC → RLP, MPFC → LLP, MPFC → RLP, and LLP → RLP). Two mutual inhibitory ECs were observed in the TBI group, between the PCC and MPFC, and between LLP and PCC, while one mutual excitatory EC was observed in the HC, between MPFC and RLP. Both groups displayed right hemispheric lateralization, particularly characterized by ECs involving the RLP and MPFC. The intrinsic connectivity of all DMN nodes in both groups displayed self-inhibition, with the strongest inhibitory value observed in the PCC for TBI, and in MPFC for the HC group. The RLP displayed the strongest outgoing ECs in both groups. Also, a statistical comparison of connectivity from the RLP → LLP reaches a near-moderate effect size, suggesting moderately stronger extrinsic excitatory EC in HC compared to the TBI group originating from RLP to LLP (*p* = 0.136, Hedge’s *g* = 0.48).

**TABLE 7:** Statistics of the endogenous connectivity parameter of the winning model.

Connection	BPA ECP		p^2^	Effect size*^b^*
	HC	TBI		
PCC	–0.8	–1.28	-	-
MPFC	–1.28	–0.71	-	-
LLP	–0.40	0.41	-	-
RLP	–0.40	–0.70	-	-
PCC→ MPFC	–0.15	–0.06	0.585	0.17
PCC → LLP	−	–0.01	0.160	0.45
PCC→ RLP	–0.09	0.02	0.439	0.24
MPFC → PCC	0.32	–0.26	0.185	0.42
MPFC → LLP	–1.28	–0.71	0.805	0.08
MPFC→ RLP	–0.19	–0.04	0.257	0.36
LLP→ PCC	0.01	–0.12	0.213	0.40
LLP→ MPFC	0.10	0.11	0.875	0.05
LLP→ RLP	–0.40	–0.41	0.687	0.13
RLP→ PCC	0.05	0.30	0.354	0.29
RLP→ MPFC	0.06	0.26	0.554	0.19
RLP→ LLP	0.40	0.16	0.136	0.48

*PCC: posterior cingulate cortex, MPFC: medial prefrontal cortex, LLP: left lateral parietal, RLP: right lateral parietal, BPA: Bayesian parameter averaging, ECP: endogenous connectivity parameter, TBI: traumatic brain injury.*

*^a^Independent sample t-test of the individual endogenous parameters from each group, thresholded at p < 0.05.*

*^b^Cohen’s corrected d (Hedge’s g).*

## Discussion

We analyzed the DMN *via* rsfMRI data in HC and TBI groups using LFF activations, ICA extraction, FC analysis, and ECs *via* cross-spectral density analysis. Our results demonstrated that compared to HC, TBI participants experienced alterations in the functional organization of the DMN. In particular, lower neuropsychological performance, decreased regional activations, lower FC values, and altered ECs were observed within the TBI group compared to HC. The neuropsychological test results have shown that the TBI group performed moderately worse than HC in the domains of general cognitive ability, verbal memory, and visual memory. Cognitive and memory deficits following TBI have been well-documented, where studies have shown impairments in verbal memory, visual memory, and cognitive domain among the TBI survivors (Caeyenberghs et al., 2014; Robb Swan et al., 2015; Huang et al., 2017; Lutkenhoff et al., 2020; Li et al., 2021). However, we did not find any significant difference between groups on other neuropsychological domains, which established the asynchrony between impaired connectivity and cognitive deficits commonly reported in the literature (van der Horn et al., 2016; Rajesh et al., 2017).

The result of LFF activations showed that the thalamus was hypoactivated in TBI compared to HC, in line with previous literature (Leung et al., 2016), particularly in patients with complaints (Grossman and Inglese, 2016). The thalamus is implicated in the regulation of awareness and consciousness and filters information between the brain and the body, essentially functioning as a relay station (Wang et al., 2014), and damage to the thalamus has been shown to affect the attention, executive function, and memory (Lutkenhoff et al., 2020). Previous studies have demonstrated the structural and FC of the thalamus with the DMN (Fransson, 2005; Cunningham et al., 2017), therefore indicating that the thalamus has a key role in DMN functions (Alves et al., 2019), especially during mindfulness and consciousness (Wang et al., 2014; He et al., 2015). Studies have also shown that the FC between the DMN nodes and thalamus was increased in TBI compared to HC in acute (Sours et al., 2015), subacute (Tang et al., 2011; Banks et al., 2016), and chronic stages (Nordin et al., 2016) that may be accompanied by increased structural connectivity (Munivenkatappa et al., 2016). Other studies found diminished FC in the thalamus in the chronic stage of TBI (Moreno-López et al., 2016; Xiong et al., 2016). However, despite the thalamic hypoactivation, we found no significant difference in FC between the nodes of the DMN and thalamus in our HC and TBI cohorts, and the effect size comparison revealed a negligible effect.

In terms of the FCs between other brain regions, the TBI group displayed significantly lower FCs between the DMN and other RSNs compared to the HC group. This finding is in contrast with previous research that indicated hyperconnectivity as a common response to TBI, especially involving network hubs in the chronic stage (Hillary et al., 2014, 2015; Hillary and Grafman, 2017; Roy et al., 2017). On that note, decreased FC in the acute and subacute stages has been established in the literature (Manning et al., 2019), while diminished FC in PCC and precuneus regions was found in the subacute mild TBI cohort (Iraji et al., 2015), corroborating our findings. In the acute stage, reduced FCs within the nodes of the DMN were also found (Dall’Acqua et al., 2017), which gradually increased and served as a compensatory mechanism. The additional connectivity recruitments culminated in the functional hyperconnectivity within the first year of recovery after the initial injury (Hillary et al., 2014), with peak hyperconnectivity at 6 months post-injury (Roy et al., 2017) before being reduced as the brain is found to be the most optimal route to balance between performance and metabolic cost (Hillary and Grafman, 2017; Roy et al., 2017). Using seed-level analysis, we found that the FC from the PCC to the MTG is significantly reduced in the HC group. The MTG corresponds to Brodmann’s area 22 and is involved in language processing. In addition, ROI-to-ROI analysis also indicated decreased FC between the DMN and LN, as well as between the DMN and FPN. Several studies have shown that TBI may impair the language domain, notably language comprehension, production, and coherence (Marini et al., 2014; Shumskaya et al., 2017; Huang et al., 2020). Hence, the impaired FC between the DMN and LN is corroborated.

Building on our findings in neuropsychology scores and the FCs, we performed correlation analysis between these two results and found significant correlations between inter-network FC and several neuropsychological domains. Our results showed that lower FCs between DMN and LN nodes significantly correlate with lower test scores in general cognitive ability and verbal memory domain in the TBI group. The verbal memory domain also subserves language components (Acheson and MacDonald, 2009; Schwering and MacDonald, 2020); thus, this correlation indicates a possible association between diminished FC involving the LN and lower test scores in the verbal memory tests. On the other hand, scores in matrix reasoning and visual memory were negatively correlated with the FC strength between PCC and visual medial node. This finding suggests that an increase in FC between the DMN and VN may affect the performance in visual memory and matrix reasoning. Additionally, correlation analysis revealed that the PCC was implicated in all significant correlation results, implying an important role played by the PCC as the network hub between the DMN and other RSNs (Hillary and Grafman, 2017).

Finally, the EC of the TBI group also showed alterations compared to HC. Hemispheric asymmetry was observed in both groups, with the ECs lateralized to the right hemisphere. The DMN is established to be asymmetrically organized, determined by the influence of the parietal regions (Almgren et al., 2018). For extrinsic ECs, our results showed that the TBI group exhibited a near moderate decrease in EC particularly from RLP to LLP, characterized by a lower excitatory EC parameter compared to the HC. In this case, the lower excitatory influence exerted by the RLP to the LLP was translated into hypoactivated left parietal areas. Moreover, the TBI group displayed more inhibitory connectivity compared to HC. This finding is in line with our previous study, which found more negative ECs in the TBI group with higher strength (Abdul Rahman et al., 2020). The extrinsic inhibitory connections are the negative influence exerted by one region to another, also known as the baseline inhibition (Stephan and Friston, 2010), due to the population of inhibitory neurons. The excess of negative ECs in TBI may signal the higher number of inhibitory neurons in brain connectivity that play a role in exerting baseline inhibitions between different regions.

Within the intrinsic ECs, all four nodes displayed self-inhibition in both groups. Greater inhibitory strengths were observed in the nodes that exert greater influence, especially in the MPFC and RLP. This observation is expected, as the dominant nodes of the network typically exhibit prolonged and uninhibited activity (Almgren et al., 2018). However, the greatest self-inhibition parameter in the TBI group was observed in PCC even though it did not have any dominant ECs originating from it. This finding may explain the lower precuneus activation that we found in the TBI group. The self-inhibition connections represent the decay rate of neural activity in each area and characterize the region’s susceptibility to the outside influence, with lesser self-inhibition indicating a region’s increased sensitivity to the inputs from other regions (Esménio et al., 2020). Therefore, lower sensitivity toward the outside influence resulted in lower activity observed in the precuneus region.

## Limitation and Conclusion

There are a few limitations to this study that should be considered when interpreting the findings. First, we rely on admission to the emergency department for recruitment of our samples, and the recent outbreak of the coronavirus pandemic has hindered us to recruit more participants; thus, our sample size remains small. Consequently, our small sample size may have precluded statistical significance on multiple comparison corrections at the cluster level. Nevertheless, while our findings are valid for the sample that we have recruited, extrapolation to the general population must be done cautiously and with the support of future studies with a bigger sample size. Based on our observation, the effect size analysis of our results suggested that a bigger sample size can lead to significant results after the correction for multiple comparisons. Second, at best, these findings are initial steps in understanding the heterogeneous nature of non-severe TBI across multiple factors, and we have explored these effects against a sample of homogeneous race and gender among the Malaysian population. Notwithstanding that, our study is reproducible to analyze other contributing factors that may change the findings observed in this study, such as the inclusion of different races and gender, or from the perspective of education and socioeconomic factors.

In conclusion, TBI resulted in the functional reorganization of the brain, from the aspect of activity and connectivity. These aberrations subsequently altered the EC of the DMN, changing the intrinsic and extrinsic influence patterns exerted by the nodes. Furthermore, lower performance within verbal memory, visual memory, and cognitive flexibility was widespread among the TBI group. Therefore, our observations suggest that these changes in brain organization and functions were linked to the debilitating effects of TBI, and this knowledge can be applied in interventional plans and recovery of TBI survivors.

## Data Availability Statement

The raw data supporting the conclusions of this article will be made available by the authors, without undue reservation.

## Ethics Statement

The studies involving human participants were reviewed and approved by Institutional Ethics Committee (IEC) of Universiti Sains Malaysia. The patients/participants provided their written informed consent to participate in this study.

## Author Contributions

AIAH, NN, HO, ZI, AHA, ARA, MR, KM, AO, ZE, NS, RK, HI, MA, KA, PV-S, ML-B, BB, JS, HY, PS, PSJ, AA, and JMA: conceptualization. AIAH, NN, and JMA: project administration. MRAR: investigation (literature reviews) and writing—original draft. MRAR, AIAH, HO, and WJC: methodology. MRAR, HO, WJC, AHA, DF, WW, MM, HU, MFMZ, SA, and ZZ: data collection. MRAR, HO, and WJC: data curation. MRAR, HO, WJC, and WW: data analysis. MRAR, AIAH, HI, AO, PV-S, ML-B, and JMA: writing — review and editing. MRAR: visualization. All authors contributed to the article and approved the submitted version.

## Conflict of Interest

The authors declare that the research was conducted in the absence of any commercial or financial relationships that could be construed as a potential conflict of interest.

## Publisher’s Note

All claims expressed in this article are solely those of the authors and do not necessarily represent those of their affiliated organizations, or those of the publisher, the editors and the reviewers. Any product that may be evaluated in this article, or claim that may be made by its manufacturer, is not guaranteed or endorsed by the publisher.

## Footnotes

1
www.fil.ion.ucl.ac.uk/spm/software/spm12

2
https://www.mathworks.com/products/matlab.html

3
https://www.nitrc.org/projects/artifact_detect

4
www.nitrc.org/projects/conn
