# Supplementary figures and images for: Alteration in the Functional Organization of the Default Mode Network Following Closed Non-severe Traumatic Brain Injury
Source: Front Neurosci. 2022 Mar 28;16:833320. doi: 10.3389/fnins.2022.833320 (PMC8995774; doi:10.3389/fnins.2022.833320)

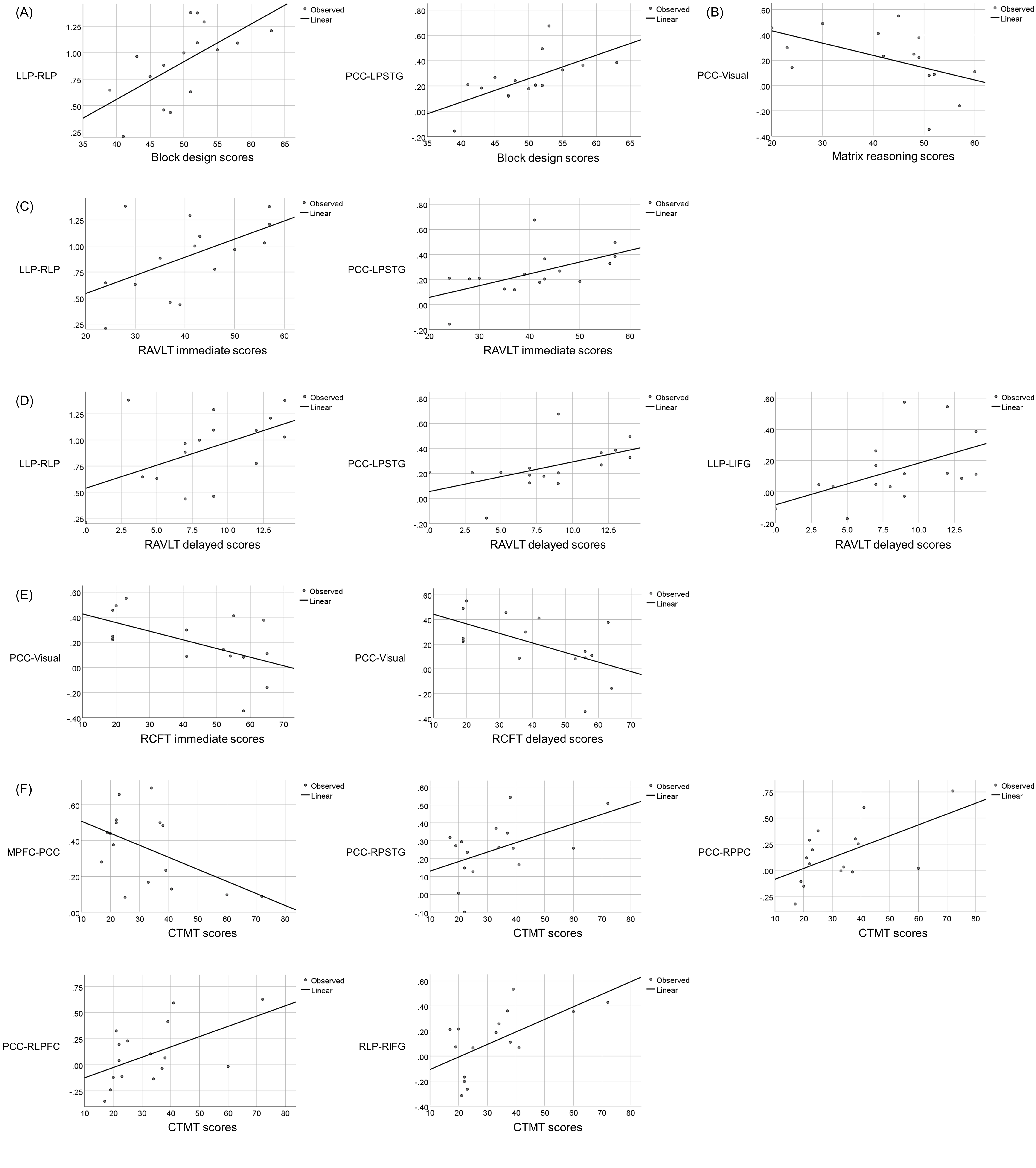

Supplement: Supplementary file 1 [file Image_1.TIF]
